# Autoluminescent *Mycobacterium tuberculosis* for Rapid, Real-Time, Non-Invasive Assessment of Drug and Vaccine Efficacy

**DOI:** 10.1371/journal.pone.0029774

**Published:** 2012-01-11

**Authors:** Tianyu Zhang, Si-Yang Li, Eric L. Nuermberger

**Affiliations:** 1 Center for Tuberculosis Research, Department of Medicine, Johns Hopkins University School of Medicine, Baltimore, Maryland, United States of America; 2 State Key Laboratory of Respiratory Disease, Center for Infection and Immunity, Guangzhou Institute of Biomedicine and Health, Chinese Academy of Sciences, Guangzhou, Guangdong, People's Republic of China; 3 Department of International Health, Johns Hopkins Bloomberg School of Public Health, Baltimore, Maryland, United States of America; University of Delhi, India

## Abstract

Preclinical efforts to discover and develop new drugs and vaccines for tuberculosis are hampered by the reliance on colony-forming unit (CFU) counts as primary outcomes for *in vivo* efficacy studies and the slow growth of *Mycobacterium tuberculosis*. The utility of bioluminescent *M. tuberculosis* reporter strains for real-time *in vitro* and *ex vivo* assessment of drug and vaccine activity has been demonstrated but a simple, non-invasive, real-time surrogate marker to replace CFU counts for real-time evaluation of drug and vaccine efficacy *in vivo* has not been described. We describe the development of a fully virulent and stable autoluminescent strain of *M. tuberculosis* and proof-of-concept experiments demonstrating its utility for *in vivo* bioluminescence imaging to assess the efficacy of new drugs and vaccines for tuberculosis in a mouse model. Relative light unit (RLU) counts paralleled CFU counts during the active phase of bacterial growth, with a lower limit of detection of approximately 10^6^ CFU in live, anesthetized mice. Experiments distinguishing active from inactive anti-tuberculosis drugs and bacteriostatic drug effects from bactericidal effects were completed in less than 5 days. The ability of a recombinant BCG vaccine to limit bacterial growth was demonstrated within 3 weeks. Use of this autoluminescent reporter strain has the potential to drastically reduce the time, effort, animals and costs consumed in the evaluation of drug activity *in vitro* and the *in vivo* assessment of drug and vaccine efficacy.

## Introduction

New tools are needed to enable development of more effective preventive and therapeutic measures to control tuberculosis (TB), which causes approximately 9 million new cases and 2 million deaths annually. More potent chemotherapeutic agents could shorten the treatment of active and latent TB infections, including those caused by multidrug-resistant strains. More effective vaccines could prevent infection or development of active TB. Efforts to discover and develop drugs and vaccines for TB are hampered by the slow growth of *Mycobacterium tuberculosis*, which requires 3–4 weeks of incubation to form colonies on solid media. Reliance on the conventional endpoint of organ CFU counts is also costly in terms of the numbers of mice, BSL-3 laboratory space and other resources needed to obtain and cultivate animal tissues at each desired time point. A reliable non-invasive, real-time method of quantifying viable *M. tuberculosis in vitro* and in live animals could dramatically increase throughput and accelerate the pace of discovery and development of new preventive and therapeutic agents.

Light production by various luciferase enzymes has been used as a real-time biomarker of bacterial viability for high-throughput screening of antibiotics and drug susceptibility testing against mycobacteria [1], [2], [3], [4], [5], [6]. The bacterial luciferases encoded by *luxAB* catalyze the oxidation of reduced flavin mononucleotide using a long-chain fatty aldehyde substrate, producing H_2_O and light (∼490 nm wavelength) in the process. Other genes of the lux operon (*luxCDE*) encode enzymes for the synthesis of the aldehyde substrate [7] . Bioluminescence produced by a strain of *Mycobacterium smegmatis* expressing *luxAB* from *Vibrio harveyi* was 2 orders of magnitude greater than that produced by a comparable strain expressing firefly luciferase [8]. Mycobacterial reporter strains expressing *luxAB* have been used to screen antibiotics and biocides *in vitro* [9] and to demonstrate proof-of-concept for evaluation of drug [3] and vaccine efficacy [8] in mice, but these constructs required the addition of exogenous substrate. Recently, Andreu *et al* [1] reported on their efforts to optimize the expression of the entire *luxCDABE* operon in mycobacteria, which culminated in non-invasive, real-time imaging of *M. smegmatis* in the lungs of mice without the need for exogenous substrate. However, efforts to create a stable autoluminescent strain of *M. tuberculosis* that could be visualized *in vivo* were unsuccessful.

Here, we report the successful engineering of *M. tuberculosis* to express the native *luxCDABE* operon from *Photorhabdus luminescens* and demonstrate proof-of-concept for rapid, serial, non-invasive, real-time evaluation of anti-tuberculosis drug and vaccine efficacy *in vivo*. The utility of such bioluminescent strains for high-throughput screening of anti-tuberculosis activity *in vitro* is also demonstrated.

## Results

### Selection of a stable and virulent autoluminescent strain of *M. tuberculosis* H37Rv (AlRv)

**Figure 1:**
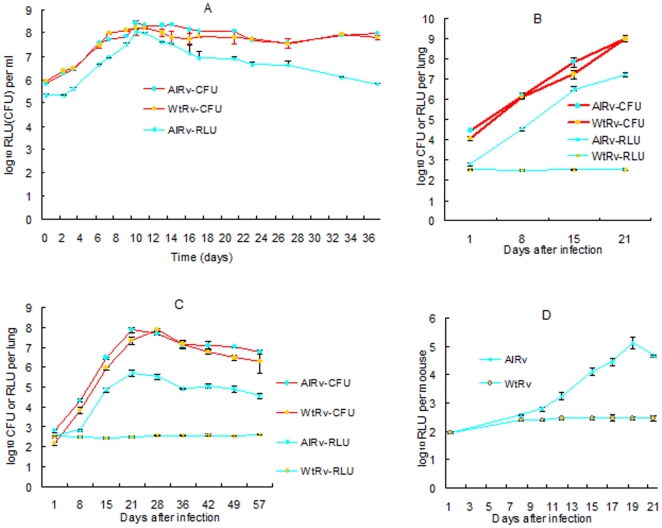
Growth comparison of WtRv and AlRv on the basis of RLU and CFU counts. *In vitro* (A), in lung homogenates from BALB/c mice after high-dose aerosol infections (implanting 4.05±0.11 and 4.44±0.05 log_10_ CFU, respectively) (B) or low-dose infections (implanting 2.15±0.08 and 2.78±0.07 log_10_ CFU, respectively) (C), and in live, anesthetized mice after high dose infection (D).

The natural *luxCDABE* operon was cloned into four different expression vectors under control of the constitutive *hsp60* promoter [10]. Although *E. coli* transformants containing each of three different integrative plasmids and the extra-chromosome vector pTYOEH were obtained, no *M. tuberculosis* clones with pTYOEH were obtained, which is consistent with a recent report [1] that stable luminescent mycobacterial clones could not be obtained using episomal vectors. Transformants containing pTYOK and pTYZOK2 exhibited strong luminescence, visible to the naked eye. The one containing pOAIK2 produced much weaker light. Clones containing pTYZOK1 or pOAIK1 did not produce light above background levels, implying that the orientation of the *luxCDABE* operon is important and that greater expression of *luxAB* with additional copies does not increase light production. The strain containing pTYOK was selected for further study because it did not contain *luxAB* in addition to the *luxCDABE* operon. This autoluminescent H37Rv (AlRv) strain grew as well as the parent strain (WtRv), both *in vitro* (Figure 1A) and after high- and low-dose aerosol infection of mice (Figure 1, B&C). High-dose infection with either strain resulted in 100% mortality between 21 and 23 days post-infection.

### Correlation of luminescence and colony-forming unit (CFU) counts *in vitro* and *in vivo*

During logarithmic growth of the AlRv strain *in vitro* (Figure 1A), relative light unit (RLU) counts paralleled CFU counts. In stationary phase, the RLU/CFU ratio declined steadily, presumably due to reduced availability of the FMNH_2_ substrate, as previously described [1]. Similar correspondence between RLU and CFU counts during logarithmic growth was observed in lung homogenates after aerosol infection (Figure 1, B&C) (r^2^ = 0.995 for high-dose infection; r^2^ = 0.978 for low-dose infection). As opposed to the *in vitro* situation, however, the RLU/CFU ratio remained similar in lung homogenates from mice receiving low-dose infection even after CFU counts peaked with the onset of acquired immunity. RLU counts in lung homogenates were at least 2 log_10_ greater than background RLU values within 8 days of high-dose infection and within 15 days of low-dose infection, providing an ample window for the assessment of bactericidal and bacteriostatic properties of antimicrobials. Most importantly, however, RLU could be detected and serially monitored in real-time in live, anesthetized mice by the second week after high-dose infection with AlRv (Figure 1D). RLU counts in these mice peaked at 2.5 log_10_ RLU above the background level before the animals succumbed to infection. In live low-dose-infected mice, RLU counts only exceeded the background value for a short window of time, when the CFU counts exceeded 6 log_10_ during weeks 3 to 6 post-infection (data not shown). Beyond that point, RLU counts did not exceed the background value despite stable CFU counts in the lungs just below 7 log_10_.

### Serial, real-time monitoring of anti-tuberculosis drug activity *in vitro*

**Figure 2:**
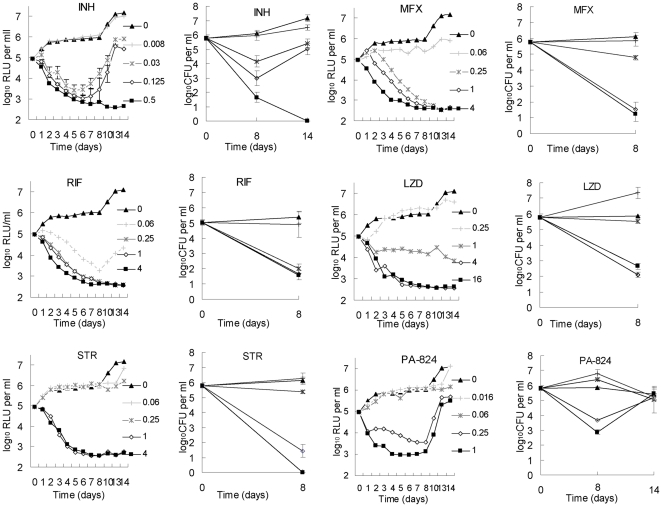
Correlation between RLU and CFU measurements during drug exposure *in vitro*. Abbreviations: INH, isoniazid; RIF, rifampin; STR, streptomycin; MXF, moxifloxacin; LZD, linezolid.

RLU counts provided a reliable real-time surrogate for estimating the inactive, bacteriostatic and bactericidal concentrations 6 anti-tuberculosis drugs with differing mechanisms of action (Figure 2). Importantly, concentrations resulting in ≥2 log reductions in RLU by 4–5 days resulted in ≥2 log reductions in CFU counts assessed after 8 days of incubation. At these bactericidal concentrations, drug activity is plainly evident in the RLU counts measured after as little as 2 days of incubation. Serial monitoring of the RLU counts also allowed for early detection of selection of resistant organisms in the case of isoniazid (INH) and PA-824, consistent with the high frequency of spontaneous mutations (≥10^−6^) conferring resistance to these two agents [11], [12].

### Serial, non-invasive, real-time monitoring of anti-tuberculosis drug efficacy *in vivo*

**Figure 3:**
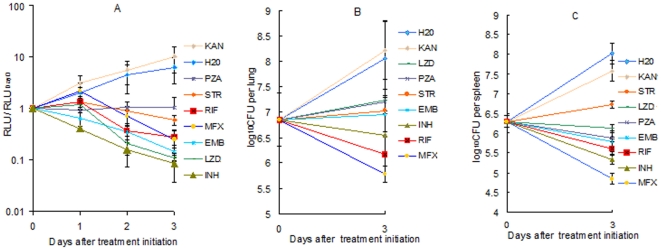
Serial, non-invasive, real-time assessment of anti-tuberculosis activity in infected mice. (A) Mean RLU count (± SD) assessed daily in live, anesthetized mice and normalized to the baseline RLU value. Mean (B) lung and (C) spleen CFU counts (± SD) at baseline and after 3 days of treatment. Abbreviations: KAN, kanamycin; STR, streptomycin; PZA, pyrazinamide; MFX, moxifloxacin; RIF, rifampin; EMB, ethambutol; LZD, linezolid; INH, isoniazid. Doses (in mg/kg): KAN 150; STR 150; PZA 150; MFX 200; RIF 40; EMB 200; LZD 200; INH 10.

RLU measured in live, anesthetized mice treated with a variety of anti-tuberculosis drugs effectively discriminated active from inactive treatments in as little as 2 days (Figure 3A). Treatment with water or kanamycin (KAN; to which the AlRv strain is resistant) permitted a nearly 10-fold increase in RLU over the first 3 days, whereas active drugs prevented any increase over baseline. The most effective agent at reducing RLU counts over the first 3 days, INH, resulted in a 10-fold decrease from baseline. When RLU counts were normalized to the pre-treatment baseline value for each mouse and group means were compared, all drugs other than KAN were significantly more active than water after just 2 days of treatment (p<0.01). Comparison of RLU counts with CFU counts from lungs and spleens after the same duration of treatment (Figure 3, B&C) confirms the potential of RLU counts as a surrogate for CFU counts in screening for anti-tuberculosis activity. As with the RLU counts, mean spleen CFU counts after 3 days of treatment showed all drugs other than KAN to be superior to water (p<0.01). On the basis of lung CFU counts, however, neither PZA nor LZD was statistically superior to water. These results illustrate the statistical power that may be gained from serial measurements in the same animal. Interestingly, the magnitude of the reductions in RLU and CFU did not always correlate precisely. For example, ethambutol (EMB) and linezolid (LZD) each reduced the RLU counts by nearly 10-fold over 3 days (comparable to INH), but had largely bacteriostatic effects on the basis of lung and spleen CFU counts. On the other hand, moxifloxacin (MFX) was somewhat less effective at reducing RLU counts over the first day of treatment, but was the most effective agent at reducing CFU counts. The reason for such discrepancies is likely mechanistic in nature, as similar patterns were observed *in vitro* for MFX and LZD (Figure 2). For example, protein synthesis inhibition by LZD may reduce luciferase or substrate synthesis or FMNH_2_ availability without cell death whereas inhibition of DNA gyrase only stops the luciferase reaction upon cell death.

### Serial, non-invasive, real-time monitoring of vaccine efficacy *in vivo*

**Figure 4:**
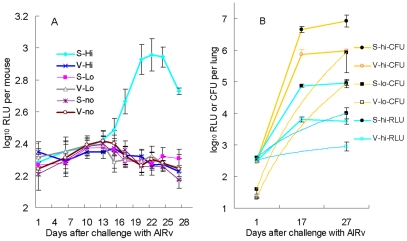
Serial, non-invasive, real-time assessment of vaccine efficacy in mice challenged with the AlRv strain of *M. tuberculosis*. (A) Mean RLU count (± S.D.) assessed in live, anesthetized mice vaccinated with either sham (S) or rBCG30 (V) vaccines followed by high-dose (hi), low-dose (lo) or no aerosol infection with the AlRv strain. (B) Mean CFU and RLU counts (± SD) from lung homogenates obtained 1, 17 and 27 days after challenge with the AlRv strain.

Aerosol infection with rBCG30 [13] implanted 2.11±0.26 log_10_ CFU/lung. Four weeks later, the high-dose and low-dose aerosol infections with AlRv implanted 2.47±0.10 and 1.47±0.19 log_10_ CFU/lung, respectively. Serial monitoring of RLU in live, anesthetized animals revealed a steady increase in RLU counts in the sham-vaccinated, high-dose-infected group, beginning 2 weeks after infection, peaking 3 weeks after infection, and remaining elevated above baseline 4 weeks after infection (Figure 4A). RLU counts in sham-vaccinated mice challenged with a high dose of *M. tuberculosis* first exceeded those in rBCG30-vaccinated mice at 13 days post-challenge (p<0.05) and the difference became highly statistically significant by 16 days post challenge (p<0.001). On the other hand, RLU counts among rBCG30-vaccinated mice which received high-dose, low-dose, and no AlRv infection could not be differentiated from those in sham-vaccinated, uninfected mice throughout the 4 weeks post-infection. The efficacy of rBCG30 vaccination was confirmed by lung CFU counts at 17 and 27 days after infection, demonstrating that vaccination restricted *M. tuberculosis* growth by roughly 1 log_10_ in both the high- and low-dose infected mice (p<0.0001). RLU counts on the lung homogenates themselves correlated very well with the CFU count results available 4 weeks later (r^2^ = 0.98) (Figure 4B).

### Stability of the autoluminescent *M. tuberculosis* strains

The AlRv strain was very stable for light production despite propagation in media without selective kanamycin. After 37 days of *in vitro* growth in broth (Figure 1A), 67 (95.7%) of 70 colonies isolated on drug-free plates retained autoluminescence. Eight weeks after mouse infection (Figure 1C), 68 (91.9%) of 74 colonies randomly selected from 3 lungs and 57 (91.9%) of 62 colonies randomly selected from 3 spleens retained autoluminescence. All colonies that lost autoluminescence also failed to produce light after the addition of exogenous substrate (1% decanal in alcohol, v/v). Finally, 30 of 30 colonies isolated after serial passage of *M. tuberculosis* H37Ra integrated with pTYZOK2 in drug-free medium 3 times over 3 months retained autoluminescence.

## Discussion

Although several imaging methods [1], [2], [3], [14], [15] have been developed for quantifying the mycobacterial burden in live animals, none of them have demonstrated the potential for daily monitoring using a simple, rapid and inexpensive method feasible enough to facilitate drug discovery and development efforts in nearly any laboratory setting. In this study, we demonstrate that a recombinant autoluminescent strain of *M. tuberculosis* expressing *luxCDABE* enables rapid, serial, non-invasive, real-time monitoring of the viable bacterial population in live mice during experimental chemotherapy or in the bacterial challenge phase after vaccination. By reducing reliance on the conventional endpoint of organ CFU counts, this advance promises to significantly reduce the time, effort and resources needed to screen new drug and vaccine candidates *in vivo*. By enabling serial imaging of the same animal, the use of this reporter strain can also reduce the numbers of animals consumed and improve statistical power. Lower compound requirements, reduced need for BSL-3 animal housing space, and improved laboratory safety through fewer opportunities for handling infected tissues and bacterial aerosolization are additional benefits. There is no need for expensive or biohazardous reagents and the only necessary equipment is a relatively inexpensive tabletop luminometer. With small sample volumes (∼200 µl) and no exogenous substrate needed, this system is very amenable to high-throughput screening. For live animal imaging, it is even possible to avoid the need for anesthesia if the luminometer is modified to restrain the mouse for the 3-second detection period. Even the most rapid *in vivo* assessments based on prevention of mortality, weight loss, lesion development or splenomegaly after high-dose challenge with *M. tuberculosis* require a minimum of 4 weeks to distinguish active from inactive drugs and 7–8 weeks for CFU counts to distinguish bactericidal from bacteriostatic treatments. Under the *in vivo* testing conditions evaluated here, 3 days of drug exposure (requiring≤10 mg of compound *in vivo*) was sufficient to discriminate active drugs from drug-free controls. The small compound requirement is important for screening compound libraries, especially for natural products or other compounds in short supply. Given the rapid, simple nature of the assay technique, we estimate that up to 500 mice could be screened per day by one person. Additional studies with classes of drugs which act by different mechanisms of action will be required before this system can be considered to be qualified for routine use.

We did not obtain any autoluminescent *M. tuberculosis* clones with an extra-chromosomal plasmid expressing *luxCDABE*, which is in agreement with a recent report [1], as well as efforts made 2 decades ago and reported in 1991 [16]. Ironically, the first integrative vector for mycobacteria was described that same year [17]. For the time being, it appears to be necessary to use integrative vectors to generate stable autoluminescent clones. Our autoluminescent strain of *M. tuberculosis* was essentially as virulent as its wild-type parent strain. The integrated *luxCDABE* operon was very stable, both *in vitro* and *in vivo* (96% and 92% retention of autoluminescence, respectively). However, it was not completely stable and the non-luminescent revertants remained so despite the addition of exogenous substrate, suggesting that the *luxCDABE* operon could possibly be excised by the L5 integrase enzyme at a very low rate [18]. The mutant ratio in the population did not change very significantly over a long period, providing further proof that the AlRv strain has a growth rate similar to the WtRv parent. Like previous authors using *luxAB*-producing luminescent strains, we found a strong and consistent correlation between RLU and CFU counts in actively multiplying bacteria and a reduction in the RLU/CFU ratio when the organisms entered stationary phase, presumably due to reduced metabolic activity.

One obvious limitation of the non-invasive method evaluated here is the large bacillary burden needed for detection. The autoluminescence of the AlRv strain could not be differentiated from the background level in live, infected mice when the AlRv population fell much below 10^6^ CFU per lung after aerosol infection. The greater bacterial burden and more widespread dissemination of infection following intravenous infection decrease the lower limit of detection somewhat, although we did not test this formally. While the modest sensitivity of the test system limits the ability to detect differences in activity between highly active compounds or regimens or to evaluate drug effects against smaller populations of persistent bacilli, a sufficient dynamic range exists for discriminating active from inactive compounds, bacteriostatic from bactericidal effects, and weak from strong bactericidal effects. Although we focused on existing anti-mycobacterial drugs in this study, the ability to serially image the same animals over time in this system provides excellent power for discriminating small effects on bacterial metabolism and viability which should make it useful for identifying early lead compounds with weaker effects. Use of immunocompromised mice could further extend the upper limit of the dynamic range of the assay [6], especially if the RLU/CFU ratio does not decline as markedly as in immunocompetent animals after the onset of the adapative immune response, but we have not examined this possibility.

Assaying autoluminescence in organ homogenates, as opposed to live mice, also improves the dynamic range of the system, as demonstrated in Figure 1. Used in this way, the dynamic range and sensitivity of our system is similar to what has been reported previously [6].

Enhancing the expression of the *luxCDABE* operon might increase the sensitivity of this reporting system. Using a stronger promoter, a multi-copy vector, a better optimized Shine-Dalgarno sequence or a codon-optimized *luxCDABE* operon more suitable for the high G+C content of mycobacteria [19] may increase the RLU/CFU ratio. We have compared the *hsp60*, G13 and MOP (Mycobacterial Optimized Promoter) promoters in front of *luxAB* in *Mycobacterium ulcerans*, another slow-growing mycobacterium [4] and found little significant difference among them. Andreu *et al* similarly found the *hsp60* promoter to drive the highest luminescence of the 3 luminescent reporters evaluated. A further effort to augment luciferase production and combine luciferases with different stability [20] here by adding *luxAB* from *V. harveyi* to plasmids containing *luxCDABE* did not significantly increase autoluminescence. Use of a *luxCDABE* operon codon-optimized for *Streptomyces coelicolor* [19], another G+C-rich organism, did not prove to be very sensitive in mycobacteria [1]. These results suggest that further increases in luciferase expression may have limited effects on amount of light produced.

The degree of luminescence may also be improved by increasing the availability of substrate. In a recent report, cloning an extra promoter in front of *luxCDE* to augment synthesis of the aldehyde substrate increased light output [1]. Complementation with a gene encoding a NADPH-dependent FMN reductase increased luminescence by 100-fold in yeast transfected with *luxAB* [21]. These may be useful strategies to increase the sensitivity of our strain as well.

In conclusion, we report the successful engineering of recombinant autoluminescent strains of *M. tuberculosis* which retain full replicative capacity *in vitro* and in mice. The experiments described demonstrate the potential use of RLU produced by such strains as a surrogate marker of bacterial viability in the serial, real-time evaluation of anti-tuberculosis activity *in vitro* and *in vivo* and of vaccine efficacy *in vivo*, including non-invasive quantification of viable bacteria in mice.

## Materials and Methods

### Ethics statement

All animal procedures were reviewed and approved by the Animal Care and Use Committee of Johns Hopkins University. Each experiment was performed once.

### Construction of autoluminescent *M. tuberculosis* strains

The *luxCDABE* operon from *P. luminescens* in pUTmini-Tn5-*luxCDABE* –Tc [22] was cut with *Bam*HI+*Pst*I and ligated into p60LUX [23] cut with the same enzymes to form pTYOEH, an extra-chromosomal plasmid with a hygromycin resistance marker. pTYOEH was cut with *Kpn*I and *Pst*I and the fragment containing *luxCDABE* under the *Hsp60* promoter [10] was inserted into pMH94 [17] cut with the same enzymes to give pluxOK. The attP:Int cassette in the plasmid pblueint was inserted into pluxOK cut with *Kpn*I and *Sca*I-HF™ to give pTYOK. pTYOEH was also cut with *Kpn*I and inserted into pMH94 and pTY60K [4] *Kpn*I sites to give pTYZOK1 and pTYZOK2, and pOAIK1 and pOAIK2, respectively (the only difference between plasmid1 and plasmid2 is the orientation of the fragment inserted). In pTYZOK, there is one copy of *luxAB* from *Vibrio harveyi* and one copy of *luxCDABE* from *P. luminescens*, each under one *Hsp60* promoter. In pOAIK1/pOAIK2, there is an additional copy of *luxB* from *V. harveyi* downstream of *luxCDABE*. In the end, one extra-chromosomal plasmid (pTYOEH) and three types of integrative plasmids (pTYOK, pTYZOK1/pTYZOK2 and pOAIK1/pOAIK2) were created and transformed into *M. tuberculosis* H37Rv and H37Ra by electroporation as described previously [4]. Several colonies from each plasmid/strain combination were picked from KAN-containing plates, homogenized in 2ml 7H9 broth with 0.05% Tween80, and incubated at 37°C, with shaking. When the OD_600 nm_ reached 0.5, luminescence was detected using a 20/20^n^ luminometer (Turner BioSystems), measuring light production over 3 sec. Strains were compared on the basis of RLU per ml of culture and the RLU/CFU ratio.

### *In vitro* growth curves of AlRv and WtRv

Autoluminescent *M. tuberculosis* H37Rv transformed with pTYOK (AlRv) was passaged once in BALB/c mice (Charles River, Germantown, MD) by intravenous injection. Autoluminescent colonies isolated on KAN-containing plates were homogenized with sterile glass beads in a 250 ml flask containing 30 ml 7H9 with Tween80. A culture of the parent H37Rv strain (WtRv) was started simultaneously. When the cultures reached an OD_600 nm_ of 0.6, the culture was diluted 150-fold in 30 ml of fresh 7H9 with Tween80 and incubated at 37°C, with shaking. At prescribed time points, 100 µl aliquots were assayed in triplicate for RLU counts and plated in serial 10-fold dilutions for CFU counts. CFU counts were determined after 4 weeks of incubation.

### *In vivo* growth curves of AlRv and WtRv and time-to-death curves of mice infected with these 2 strains

After mouse passage as described as above, 10 ml of undiluted broth culture and 10 ml of a 100-fold diluted culture were used to infect 95 4-to-6-week-old female BALB/c mice by aerosol infection using the Inhalation Exposure System (Glas-Col, Terre Haute, IN). Three mice per group were killed the day after infection and weekly thereafter for 8 weeks. The mice were first anesthetized by isoflurane inhalation and the RLU count was determined by laying the breast of the mouse over the detection hole of the luminometer and measuring light production for 3 sec for 3 times (Figure S1). The mice were then euthanized. The lungs and spleens were removed aseptically, washed in 14 ml PBS, and homogenized in 2 ml PBS (lungs) or 1 ml PBS (spleens). A 0.5 ml aliquot of homogenate was used for RLU detection. The remaining 0.5 ml of was serially diluted and plated on Middlebrook 7H11 agar. CFU counts were determined after 4 weeks incubation in 37°C. Eight additional mice in the high-dose group for each strain were held to determine time-to-death. The mice were euthanized as soon as they appeared moribund.

### Serial, real-time monitoring of anti-tuberculosis drug activity *in vitro*

The activity INH, RIF, STR, MFX, LZD, and PA-824 was assessed over a range of 4-fold increasing concentrations prepared in 1 ml of 7H9 broth to which 0.2 ml of a 1/100 dilution of AlRv broth culture (OD_600 nm_ ∼0.8) grown in 7H9 broth +0.05% Tween80 was added. RLU counts were determined daily, in triplicate, for 8–14 days. After 0 and 8 days (and after 14 days for INH and PA-824), 0.5 ml aliquots of serial 10-fold dilutions were plated on 7H11 and incubated at 37°C for up to 28 days.

### Serial, non-invasive, real-time monitoring of anti-tuberculosis drug activity *in vivo*

A log-phase culture of AlRv adjusted to an OD_600 nm_ of 0.1 was used to infect 5-to-6-week-old female BALB/c mice by tail vein injection. The day after infection (day 0), RLU counts were determined, in triplicate, under anesthesia for all mice, as described above. Forty mice with similar RLU readings were randomly allocated to treatment groups (4 mice/group) and individually marked. The treatment groups received: water alone (negative control), KAN (150 mg/kg) (a second negative control), INH (10 mg/kg), RIF (40 mg/kg), PZA (150 mg/kg), EMB (200 mg/kg), STR (150 mg/kg), MFX (200 mg/kg), or LZD (200 mg/kg). STR and KAN were administered by subcutaneous injection in 0.2 ml sterile PBS. The remaining drugs were prepared and administered by gavage in 0.2 ml as previously described [24], [25], [26], [27]. Treatment was administered on days 0, 1 and 2. RLU were detected under anesthesia daily. Mice were killed on day 0 and day 3 after to determine lung and spleen CFU counts as described above.

### Rapid, serial, non-invasive, real-time monitoring of vaccine efficacy in mice challenged with *M. tuberculosis*

5-to-6 week-old female BALB/c mice were vaccinated by the aerosol route with either (i) a broth culture (OD_600 nm_ of 0.25) of a recombinant BCG strain over-expressing antigen 85B (rBCG30 or V), or (ii) 7H9 broth only (Sham or S), using the Inhalation Exposure System. The day after vaccination, 4 mice from the former group were killed to determine the number of rBCG30 implanted. Four weeks later, 12 mice from each vaccination group were aerosol-infected with a 50-fold dilution (hi) of a broth culture of the AlRv strain (original OD_600 nm_ of 0.6). Another 12 mice from each group were infected with a 500-fold dilution (lo) of the original culture. Additional mice were vaccinated but not challenged with AlRv (no). The day after infection with AlRv, 4 mice from each of these groups were killed and the lung homogenates were plated on 7H11 plates containing KAN at 25 µg/ml (to select for AlRv) and plates containing hygromycin at 40 µg/ml (to select for rBCG30) for CFU count determination. RLU counts were determined under anesthesia on days 1, 6, 10, 13, 15, 17, 20, 22, 24 and 27 after AlRv infection. Four mice in V-hi and S-hi groups were killed for RLU and CFU counts from lung homogenates at day 17 (the second consecutive time point at which the live RLU counts were significantly different (*P*<0.05 by *t* test)). All remaining AlRv-infected mice were killed for RLU and CFU counts from lung homogenates at day 27.

### Statistical analysis

Group means were compared using *t* tests or one-way ANOVA with Dunnett's post-test, controlling for multiple comparisons in each case. Prism 5 (GraphPad Software, San Diego, CA) was used for all analyses.

## Supporting Information

Figure S1 **Detection of RLU from live mice using the Turner BioSystems 20/20^n^ luminometer.** (A) An anesthetized mouse is placed in prone position onto reader with limbs spread out to ensure maximum exposure to chest area. (B) The unit is closed and covered with black cloak to prevent any outside light contamination.
(TIF)
