# Supplementary figures and images for: Autoluminescent Mycobacterium tuberculosis for Rapid, Real-Time, Non-Invasive Assessment of Drug and Vaccine Efficacy
Source: PLoS One. 2012 Jan 11;7(1):e29774. doi: 10.1371/journal.pone.0029774 (PMC3256174; doi:10.1371/journal.pone.0029774)

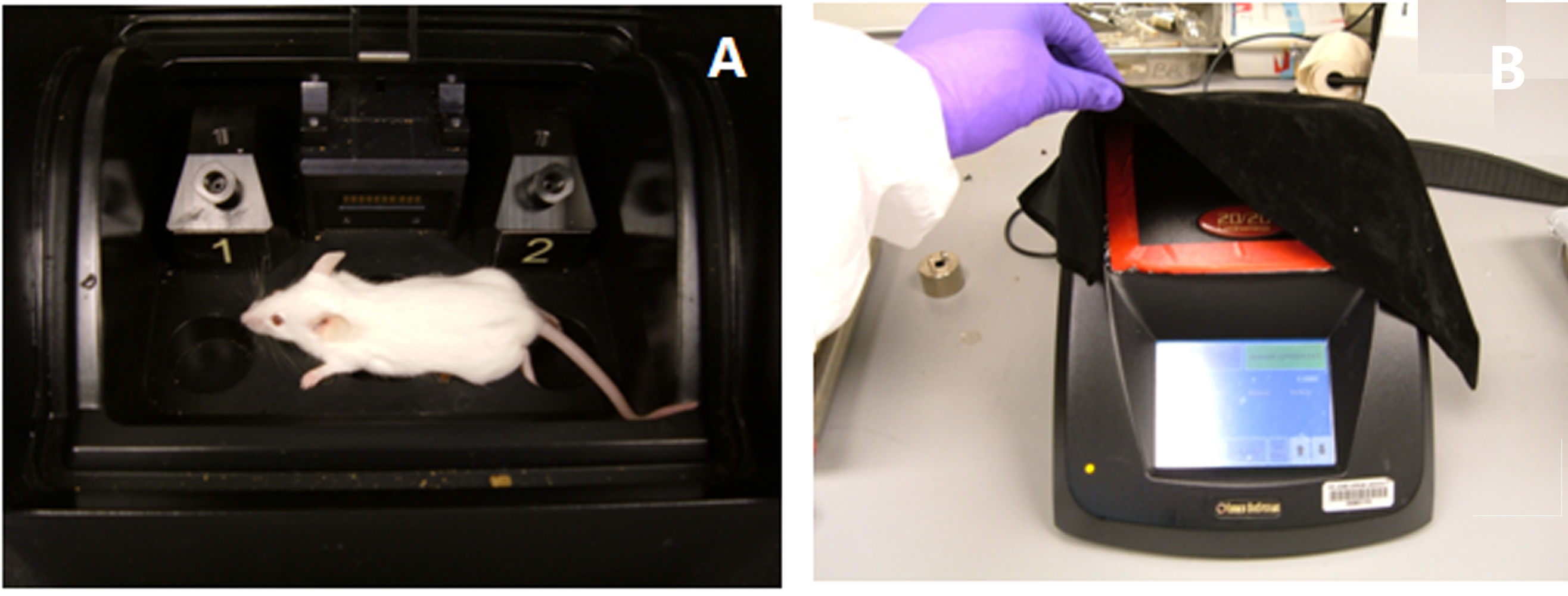

Supplement: Figure S1 — Detection of RLU from live mice using the Turner BioSystems 20/20n luminometer. (A) An anesthetized mouse is placed in prone position onto reader with limbs spread out to ensure maximum exposure to chest area. (B) The unit is closed and covered with black cloak to prevent any outside light contamination. (TIF) [file pone.0029774.s001.tif]
